# Hürden im Versorgungspfad der Demenzdiagnostik: Framework-Analyse des Projektes „iCreate“

**DOI:** 10.1007/s00391-025-02507-4

**Published:** 2025-10-17

**Authors:** Carolin Rosendahl, Kristin Rolke, Judith Tillmann, Alexander Hanke, Michael Wagner, Leon Nissen, Lara Reimer, Florian Schweizer, Philipp Schaper, Jochen René Thyrian, Klaus Weckbecker

**Affiliations:** 1 Institut für Allgemeinmedizin und Ambulante Gesundheitsversorgung, Lehrstuhl I Allgemeinmedizin und Interprofessionelle Versorgung, Universität Witten/Herdecke, Alfred-Herrhausen-Str. 50, 58448 Witten, Deutschland; 2 Klinik für Alterspsychiatrie und kognitive Störungen, Universitätsklinikum Bonn, Sigmund-Freud-Straße 25, 53125 Bonn, Deutschland; 3 Institut für Digitale Medizin, Universitätsklinikum Bonn, Venusberg-Campus 1, 53127 Bonn, Deutschland; 4 Department Psychologie, Psychologische Alternsforschung, Universität Siegen, Adolf-Reichwein-Str. 2a, 57076 Siegen, Deutschland; 5 Deutsches Zentrum für Neurodegenerative Erkrankungen (DZNE) Rostock/Greifswald, Ellernholzstr. 1–2, 17489 Greifswald, Deutschland; 6 Institut für Community Medicine, Universitätsmedizin Greifswald, Ellernholzstr. 1–2, 17489 Greifswald, Deutschland

**Keywords:** Kognitive Beeinträchtigungen, Tablet-basierte Diagnostik, Früherkennung, Hausarztpraxis, Gedächtnisambulanz, Cognitive impairment, Tablet-based diagnostics, Timely detection, Family practice, Memory clinic

## Abstract

**Hintergrund:**

In Deutschland leben ca. 1,8 Mio. Menschen mit Demenz, Tendenz steigend. Demenz ist unterdiagnostiziert. Obwohl Hausärzt*innen erste Anlaufstelle bei kognitiven Problemen sind, erfolgt eine Diagnose oft spät und meist in Fachzentren. Das Projekt „iCreate“ untersuchte ein optimiertes Case Finding mittels Tablet-basierter Kognitionstestungen. Patient*innen mit auffälligen Ergebnissen wurden an eine Gedächtnisambulanz (GDA) zur Differenzialdiagnose überwiesen. Trotz positivem Feedback zu Praxistauglichkeit traten verschiedene Hürden im Versorgungspfad des Projektes auf. Diese werden im Weiteren kritisch beleuchtet und mögliche Lösungsansätzen zur Verbesserung eines strukturierten Früherkennungsprozesses von Demenz in der hausärztlichen Versorgung aufgezeigt.

**Methode:**

Sekundäranalyse von 21 qualitativen Interviews und 56 Projektprotokollen (Telefonnotizen, E‑Mail-Korrespondenzen, Teammeetingprotokolle) aus dem Projekt „iCreate“ mittels Framework-Analyse nach Spencer et al. [16].

**Ergebnisse:**

Im Versorgungspfad zeigten sich als Hürden: a) Patientenauswahl und -ansprache: erfolgten aus Angst vor Stigmatisierung häufig zurückhaltend, b) Zeit- und Ressourcenmangel in Hausarztpraxen und der GDA sowie c) nichtstandardisiertes Vorgehen und organisatorische Defizite im Versorgungspfad (z. B. mangelnde Kommunikation, schwierige Terminvereinbarungen) und d) eine hohe „No-Show“-Quote von ca. 28 % der überwiesenen Patient*innen bei Diagnostikterminen in der GDA.

**Schlussfolgerung:**

Die identifizierten Hürden zeigen Handlungsbedarf für eine strukturierte, patientenorientierte Demenzdiagnostik. Notwendig sind standardisierte Prozesse, interdisziplinäre Zusammenarbeit und Schulungen zur sensiblen Kommunikation.

## Abstract (en)

**Background:**

In Germany around 1.8 million people live with dementia, with the numbers rising. Dementia is underdiagnosed. Although family practitioners (FP) are often the first point of contact for cognitive problems, the diagnosis is frequently delayed and mostly made in specialized centers. The “iCreate” project examined an optimized tablet-based case-finding approach to testing cognition. Patients with conspicuous results were referred to a memory clinic (MC) for differential diagnostics. Despite positive feedback regarding practical feasibility, various barriers in the care pathway of the project were identified. These are critically examined and possible solutions for improving a structured early detection process for dementia in the FP care are presented.

**Method:**

Secondary analysis of 21 qualitative interviews and 56 project protocols (telephone notes, email correspondences, team meeting protocols) from the “iCreate” project using framework analysis according to Spencer et al. [16].

**Results:**

Key barriers identified along the care pathway included: a) patient selection and approach were frequently restrained due to fear of stigmatization, b) lack of time and resources in FP practices and the MC, c) non-standardized procedures and organizational deficiencies in the care pathway (e.g., poor communication, difficult appointment scheduling) and d) high “no-show” rate of ca. 28% of the referred patients for diagnostic appointments in the MC.

**Conclusion:**

The identified barriers highlight the need for action toward a structured and patient-centered dementia diagnostic process. Standardized procedures, interdisciplinary collaboration and training on sensitive communication are necessary.

## Hintergrund und Fragestellung

Demenzerkrankungen sind eine große Herausforderung für das Gesundheitswesen. In Deutschland leben ca. 1,8 Mio. Menschen mit Demenz [4], erwartetet werden bis zu 2,8 Mio. Menschen im Jahr 2050. Schätzungen zufolge werden weniger als 10 % aller Patient*innen mit prodromaler und etwa 40 % mit manifester Demenz spät oder gar nicht diagnostiziert [9, 10], da Diagnosen meist Fachzentren vorbehalten sind. Dies beeinträchtigt Behandlungsmöglichkeiten und das Outcome erheblich. Eine rechtzeitige Diagnostik kann helfen, (therapeutische) Optionen besser zu nutzen, die Lebensqualität zu verbessern und Angehörige zu entlasten [8]. Ein systematischer Review von 2024 hebt die verbesserte Demenz-Früherkennung als eines der vier wichtigsten Versorgungsthemen hervor [11], um die Ursachen kognitiver Defizite zu verstehen, sich mit der Erkrankung auseinanderzusetzen und Lebensanpassungen (Patientenverfügungen, Wohn- und Pflegesituationen) vorzunehmen [25]. Die Diagnosestellung wird von rund der Hälfte der Angehörigen als Erleichterung empfunden [24]. Angesichts neuer Therapieoptionen bei Alzheimer-Demenz, wie Antikörpertherapien gegen Amyloidablagerungen, gewinnt eine rechtzeitige Diagnostik weiter an Bedeutung, da sie das Behandlungspotenzial optimieren kann. Um dies zu erreichen, müssen Hürden erkannt und überwunden werden. Dieser Beitrag zeigt projektspezifische Hürden aus dem vom Bundesministerium für Gesundheit geförderten Projekt „iCreate – Digital unterstütztes Case-Finding zur Verbesserung der Diagnose und Versorgung von Patient*innen mit Demenz in der primärärztlichen Versorgung“ (ZMII2-2523FEP40) und mögliche Lösungsansätze auf. Ziel des Kooperationsprojektes „iCreate“ der Universität Witten/Herdecke, des Universitätsklinikums Bonn und des Deutschen Zentrums für Neurologische Erkrankungen (DZNE) Rostock/Greifswald war es, ein digital unterstütztes Case-Finding zur Verbesserung der Diagnostik von Demenzpatient*innen in der hausärztlichen Versorgung zu erproben. In 10 Hausarztpraxen in Bonn und dem Rhein-Sieg-Kreis nahmen 313 Patient*innen an einem Tablet-basierten Kognitionstest, durchgeführt von Medizinischen Fachangestellten (MFA), teil und erhielten bei auffälligen Testergebnissen eine weiterführende Demenzdiagnostik in der Gedächtnisambulanz (GDA) Bonn. Ergebnisse zeigen [17, 19], dass der Kognitionstest in die Versorgung integrierbar, akzeptiert und technisch umsetzbar ist. Gleichzeitig wurden projektspezifische Hürden im gesamten Versorgungspfad deutlich; diese werden in einer Sekundäranalyse aufbereitet und im Weiteren kritisch diskutiert. Vor diesem Hintergrund stellen sich die Fragen: Welche projektspezifischen Hürden beeinflussen die Demenzdiagnostik in der hausärztlichen Versorgung und wie können digitale Case-Finding-Ansätze zu deren Überwindung beitragen?

## Studiendesign und Untersuchungsmethoden

Zur systematischen Erfassung und Analyse der im Projekt „iCreate“ identifizierten Hürden wurde eine Framework-Analyse nach Spencer et al. [16] als Sekundäranalyse durchgeführt. Diese eignet sich besonders für praxisorientierte Studien mit multiplen, heterogenen Datenquellen und einem klaren Anwendungsbezug. In die Analyse wurden 21 qualitative, semistrukturierte und leitfadengestützte Interviews mit Hausärzt*innen (*n* = 7), MFA (*n* = 8) und Patient*innen (*n* = 6) aus dem Projekt „iCreate“ einbezogen. Diese wurden in einer Primäranalyse hinsichtlich der Akzeptanz und Umsetzbarkeit ausgewertet und bereits publiziert [17]. In einer darauf aufbauenden Sekundäranalyse wurden die Interviews erneut von zwei Autoren mit dem Ziel analysiert, Hinweise auf Hürden systematisch zu erfassen und zu strukturieren. Ergänzend wurden 56 Projektprotokolle (Telefonnotizen, E‑Mail-Korrespondenzen, Teammeetingprotokolle) einbezogen, die ebenfalls Hürden beschrieben und so eine breitere Kontextualisierung ermöglichten.

Die Framework-Analyse bestand aus 6 Schritten [7]:

1. Familiarisierung: sorgfältiges Lesen aller Datenquellen, Identifikation zentraler Themen und wiederkehrender Inhalte,
2. Entwicklung eines Kategoriensystems: Bildung deduktiver Oberkategorien, basierend auf ersten Eindrücken,
3. Indexierung: Codierung des gesamten Datenmaterials mit Oberkategorien und Ausdifferenzierung von induktiven Subkategorien,
4. Charting: strukturierte Zusammenfassung der codierten Inhalte in thematischen Matrizen,
5. Mapping und Interpretation: fall- und themenübergreifende Analyse zur Identifikation zentraler Hürden,
6. Zusammenführung und Bewertung: Bündelung, Reflexion und Einordnung der Hürden im Kontext des Versorgungspfades der Demenzdiagnostik.

Durch dieses Vorgehen konnte ein differenziertes und kontextnahes Verständnis der Hürden erzielt werden, das die Perspektiven von Hausärzt*innen, Patient*innen, MFA sowie der Projektmitarbeitenden in der GDA berücksichtigt.

## Ergebnisse

**Abb. 1:**
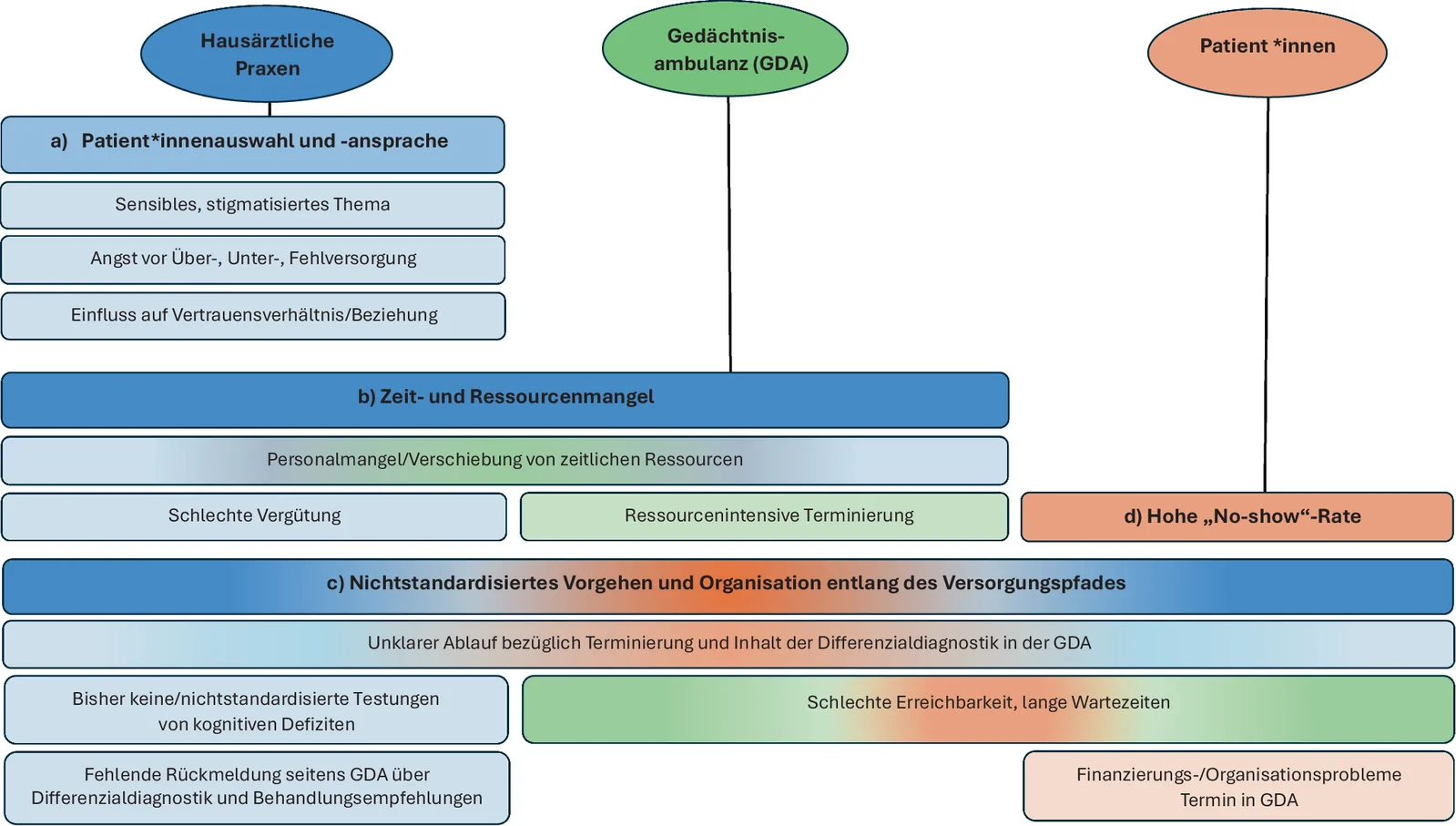
Identifizierte Hürden im „iCreate“ Projekt bei allen Beteiligten

Die Framework-Analyse ordnet die identifizierten Hürden nicht den einzelnen Beteiligten, sondern entlang des Versorgungspfades zu (Abb. 1). Identifizierte Schnittstellen sind dabei die Hausarztpraxen, die Gedächtnisambulanz (GDA) und die Patient*innen.

### Hausärztliche Praxen

#### Patientenauswahl und -ansprache

Die Ansprache von Patient*innen war für die teilnehmenden Versorgenden herausfordernd, da Demenz als sensibles und stigmatisiertes Thema gilt. Sie befürchteten, durch aktive Ansprache Ängste zu erzeugen und die Beziehung zu den Patient*innen zu belasten. Deshalb wurde vorwiegend auf die intrinsische Motivation der Patient*innen gesetzt und die Rekrutierung passiv über Aushänge in den Praxen realisiert. Ein weitere Hürde war die Auswahl geeigneter Patient*innen, um Über‑, Unter- oder Fehlversorgung zu vermeiden und ressourcenschonend zu handeln.

„Denn unser Alltag besteht häufig daraus, dass wir, sage ich mal, in diesem Niedrigprävalenzbereich, in dem wir arbeiten, halt relativ gezielt versuchen, diejenigen herauszufiltern, die tatsächlich unsere Aufmerksamkeit benötigen.“ (Ärzt*in 01)

### Hausärztliche Praxen und Gedächtnisambulanz

#### Zeit- und Ressourcenmangel

Chronischer Zeitmangel und Personalknappheit wurden im hausärztlichen und spezialisierten Setting vielfach genannt. Obwohl die Testung durch MFA gut durchführbar war und die Ärzt*innen entlastete, verschob sich das Zeitdefizit auf die MFA. Sie waren durch die Testungen zeitlich gebunden und ihre Kapazitäten fehlten somit an anderer Stelle im Praxisalltag. Auch in der GDA standen zeitweise nur wenige Termine für eine ausführliche neuropsychologische Untersuchung nach Goldstandard bei einer langen Warteliste zur Verfügung. Fehlende finanzielle Mittel für zusätzliches Personal und eine unzureichende Vergütung für kognitive Diagnostiken erschweren die langfristige Implementierung und stellen eine große Hürde dar.

### Hausärztliche Praxen, Gedächtnisambulanz und Patient*innen

#### Versorgungspfad und Organisation

Die Ressourcenknappheit wurde auch als ursächlich für die entweder fehlende oder nichtstandardisierte Testung von kognitiven Störungen im hausärztlichen Setting benannt. Je nach Praxis erfolgte direkt eine Weiterleitung zum*r Fachärzt*in oder ein kurzer Test wie der Uhrentest, um eine erste Einschätzung zu erhalten. Aber auch das Fehlen eines geeigneten Testinstrumentes wird als Hindernis angesehen. Viele Tests sind langwierig oder nicht spezifisch genug. Der eingesetzte Tablet-basierte MoCA wurde als gute Lösung wahrgenommen, trotz technischer Probleme wie instabilem WLAN oder fehlerhafter Datenspeicherung. Diese wurden insgesamt jedoch als geringfügig eingestuft.

Die fehlende automatisierte Übertragung von Testergebnissen in Patientenverwaltungssysteme hingegen erforderte zusätzliche Dokumentation und band Zeitressourcen. Auch die Schnittstelle zur GDA war hinderlich, da Prozesse unklar waren und regelmäßig Rückfragen zur weiteren Vorgehensweise auftraten (z. B. ob ein MRT vorab notwendig ist, oder wie und durch wen die Terminvereinbarung erfolgt). Zudem gab es Schwierigkeiten bei der Kontaktaufnahme und Terminvereinbarung mit der GDA, was den organisatorischen Aufwand erhöhte.

„Das hat sich ein bisschen schwer gestaltet. Also, zum einen telefonisch. Da gibt es ja nur einen kurzen Zeitraum, wo man da anrufen kann. Und wenn man dann auch nicht an jedem Morgen oder Vormittag eben hier ist, ist es etwas schwer. Und auch der E‑Mail-Kontakt war dann etwas erschwert. Also, wenn das funktionieren würde, dass man da so von eben Hausarztpraxis und Ambulanz irgendwie besser vernetzt wäre, das wäre schon auch gut, ja.“ (Ärzt*in 10)

„Leider sehr hohe Auslastung in GDA, nur noch 1 Slot in GDA pro Woche für Projekt „iCreate“ vorhanden“ (Projektnotiz, Jour fixe, Studienteam)

Aber auch für die Mitarbeitenden in der GDA gestaltete sich die Organisation schwierig. Sie notierten einen hohen zeitlichen Aufwand, mehrfach ohne Erfolg, die überwiesenen Patient*innen zu erreichen.

Fehlende oder verspätete Rückmeldungen seitens der GDA über Diagnostikfortschritt und Versorgungsempfehlungen wurde ebenfalls als hinderlich empfunden. Dies erschwerte die Weiterversorgung im hausärztlichen Setting, da häufig unklar blieb, welche Diagnostik durchgeführt wurde, und welche Behandlungsschritte folgen sollen.

„Aus meiner Sicht einfach nur eine kurze Rückmeldung darüber. Da reichen aus meiner Sicht halt auch einfach nur ein ganz einfacher Briefkopf, der Name des Patienten, und dass erwähnt wird, welche zusätzliche Diagnostik gemacht wurde bei dem Patienten, und was daraus dann letztendlich resultiert. Ein Befund. Oder aber eben auch eine Therapie oder ein Behandlungshinweis an uns. Das würde aus meiner Sicht erstmal völlig ausreichen. Eventuell auch einen Vorschlag für eine Medikation.“ (Ärzt*in 03)

### Patient*innen

#### „No shows“

Mehr als ein Viertel (28 %) der Patient*innen mit auffälligen Testergebnissen nahm keine Termine zur Differenzialdiagnostik in der GDA wahr. Gründe dafür waren u. a. die beschriebenen Hürden wie der unklare Versorgungspfad, Probleme bei der Terminvereinbarung oder fehlende Unterstützung bei der Organisation des Transports.

„Dass man einem einfach auch mal erklärt, was ist die GDA? Was macht die mit einem? Was muss man da machen? … Aber wäre schon besser, wenn man weiß, so, du musst jetzt dahin, und da passiert das und das.“ (Patient*in 05)

Ein Teil der Patient*innen konnte die notwendigen Schritte aufgrund kognitiver Beeinträchtigungen möglicherweise nicht selbstständig durchführen. Aber auch finanzielle Aspekte spielten eine Rolle, da einige Patient*innen keine Unterstützung von Angehörigen hatten und die Transportkosten nicht von der Krankenkasse übernommen wurden.

## Diskussion

Die Ergebnisse zu erlebten Hürden bieten wertvolle Einblicke in die komplexen Herausforderungen der rechtzeitigen Demenzdiagnostik mit denen Versorgende und Patient*innen im Rahmen des Projekts konfrontiert waren. Obwohl regional und in der Teilnehmendenzahl begrenzt, lassen sich zentrale Erkenntnisse mit Blick auf aktuelle Studien und Versorgungstrends auf den bundesweiten Kontext adaptieren. Daten aus der Versorgungsforschung zeigen, dass die strukturelle Unterdiagnostik von Demenz ein deutschlandweites Phänomen darstellt, trotz hoher Prävalenz und relevanter Folgen für die Versorgung [10]. Gleichzeitig wird die zentrale Rolle der Hausarztpraxis als erste in der Diagnostikkette betont [3]. Die im Projekt identifizierten Hürden verdeutlichen den Bedarf an praktikablen, alltagstauglichen Lösungen für den flächendeckenden Einsatz und spiegeln teilweise den Versorgungsalltag in Hausarztpraxen in Deutschland wider.

### Patientenauswahl und -ansprache

Eine zentrale Herausforderung war die Ansprache von Patient*innen. Kognitive Defizite und Demenz gelten als stigmatisiert, was eine offene Kommunikation erschwert. Obwohl die Vorteile eines proaktiven Ansprechens in der Literatur [22] betont werden, besteht im Praxisalltag Zurückhaltung aus Sorge, das Vertrauen zu beeinträchtigen. Diese Sorge wirkt sich negativ auf Diagnostik und Versorgung aus und verweist auf die tiefgreifende Stigmatisierung kognitiver Erkrankungen. Um dem zu begegnen, braucht es einen ganzheitlichen Ansatz [22], beginnend mit Schulungen im Grundstudium und der Facharztweiterbildung bzw. in der Ausbildung auch von MFA. Durch gezielte Trainings im empathischen, proaktiven Umgang mit sensiblen Themen wie Demenz könnten Versorgende Sicherheit im Gespräch gewinnen und Einblick in die Perspektive der Betroffenen und Angehörigen erhalten [27]. Gespräche und aktive Ansprache sollten geübt werden [15]. Eine Studie zum Ansprechen von Todeswünschen als ebenfalls sensibles und stigmatisiertes Thema zeigt, dass entsprechende Schulungen komplexe, aber bereichernde Erfahrungen ermöglichen [5]. Die Angst vor Vertrauensverlust konnte dort widerlegt werden und ein vergleichbarer Umgang mit Demenz erscheint übertragbar. Offene Gespräche, ergänzt um strukturierte Informationen zu Unterstützungsangeboten, könnten Unsicherheiten reduzieren und die Lebensqualität steigern. Auch die Quote von Fehl- oder Überversorgung und somit unnötige Überweisungen zur GDA ließe sich so verringern [20].

### Zeit- und Ressourcenmangel

Durch optimiertes Case-Finding könnte dem Problem der Zeit- und Personalknappheit begegnet werden. Zwar wurde der Tablet-basierte Kognitionstest als praktikabel bewertet, insbesondere durch die Delegation an MFA [17], jedoch zeigte sich auch, dass dies lediglich eine Umverlagerung der Arbeitsbelastung darstellte. Eine grundlegende Verbesserung erfordert strukturell verankerte Lösungen. Ein vielversprechender Ansatz ist der Einsatz digitaler Selbsttests. Erste Reviews [6] zeigen eine vergleichbare diagnostische Genauigkeit wie ärztlich durchgeführte Test und bestätigen das Potenzial solcher Tests im Primärversorgungsumfeld. Sie können ein niedrigschwelliges, ressourcenschonendes Screening ermöglichen, etwa vor dem Arztbesuch oder im Wartebereich. Ein automatisiertes Auswertungssystem könnte eine schnellere Rückmeldung liefern, jedoch besteht Forschungsbedarf hinsichtlich der Langzeitwirkung und Akzeptanz der Versorgenden und Patient*innen, Standardisierungs- und Umsetzungspotenzial. Ziel sollte die jedoch routinierte Integration kognitiver Tests ohne zusätzliche Belastung für Praxisteams sein. Dies könnte zudem gezieltere Überweisungen ermöglichen und somit auch die Versorgenden im Fachsetting entlasten.

### Nichtstandardisiertes Vorgehen

Ein Hindernis war das Fehlen eines standardisierten Vorgehens. In den Praxen wurden Tests unsystematisch oder gar nicht durchgeführt. Dies ließ sich auf den Mangel an praxistauglichen Verfahren und deren Integration zurückführen. Auch die Literatur beschreibt dies. Wangler et al. [28] betonen, dass viele Instrumente zu zeitaufwendig oder ungenau für die Primärversorgung sind, was zu einer Zurückhaltung bei der Anwendung führt. Das MoCA wurde als geeignetes Instrument gesehen. Vorteile waren reduzierte Dokumentationslast und die strukturierte Durchführung. Die technischen Probleme wie instabiles WLAN oder fehlerhafte Datenspeicherung verdeutlichten den Bedarf an robusteren, offline-fähigen Systemen. Gesundheitspolitisch braucht es daher verbindliche Leitlinien zur Demenzfrüherkennung. Diese sollten Empfehlungen zu Indikation, Testauswahl sowie weiterem Vorgehen enthalten und könnten die diagnostische Sicherheit und Versorgungsqualität erhöhen.

### Unklarer Versorgungspfad und organisatorische Hürden

Technische und organisatorische Hürden erschwerten die intersektorale Zusammenarbeit. Obwohl Testergebnisse automatisiert ausgewertet wurden, war eine manuelle Weiterleitung nötig, ein zeitaufwendiger und fehleranfälliger Prozess. Auch unklare Abläufe sowie lange Wartezeiten in der GDA trugen zur Fragmentierung bei. Diese Herausforderungen sind nicht neu [1]. Studien beschreiben die Intransparenz und mangelnde Koordination im deutschen Gesundheitssystem als zentrale Barrieren für patientenzentrierte Versorgung [21]. Die elektronische Patientenakte (ePA) könnte hier helfen. Sie kann den Austausch medizinischer Daten vereinfachen und standardisieren. Durch eine digitale Ablage von Testergebnissen und Befunden lassen sich Informationsverluste vermeiden, die Behandlungsqualität verbessern und unnötige Doppeluntersuchungen verhindern. Zwar ist die Implementierung der ePA bisher schwierig, dennoch bleibt sie langfristig zentral für eine vernetzte Versorgung [13]. Ergänzend braucht es standardisierte Überweisungspfade mit klaren Kriterien, um Missverständnisse zu reduzieren.

### „No Shows“ und Hürden in der Umsetzung der weiterführenden Diagnostik

Auffällig war die Zahl an „No Shows“, Patient*innen mit auffälligem Testergebnis, die den Termin in der GDA nicht wahrnahmen. Gründe reichten von langen Wartezeiten, fehlender Begleitung oder unklarer Terminvergabe bis zu finanziellen und psychologischen Barrieren. Diese Ergebnisse decken sich mit der aktuellen Literatur [12, 23]. Um Abbrüche zu reduzieren, braucht es eine systematische Erhebung individueller Barrieren, etwa durch Fragebögen oder Gespräche mit den Betroffenen und ihren Angehörigen. So können Probleme wie Mobilität, Sprachbarrieren oder Unterstützungsbedarf früh erkannt werden. Auch der Wunsch nach keiner weiteren Diagnostik und das Recht auf Nichtwissen sollten explizit berücksichtigt werden. Menschen mit Demenz dürfen Diagnostik und Behandlung ablehnen [26]. Dies sollte respektvoll in die Versorgungsplanung einbezogen und als eigenständige Hürde im Versorgungspfad verstanden werden. Die partizipative Einbindung der Patient*innen und eine gemeinsame Entscheidungsfindung sollten von Beginn an im Vordergrund stehen [14]. Hierfür bietet sich das bio-psychosoziale ICF-Modell der WHO an, das persönliche, soziale und gesellschaftliche Faktoren berücksichtigt. Fehlende Unterstützung, familiäre Belastung oder finanzielle Unsicherheit lassen sich so rechtzeitig erkennen und adressieren. Auch eine gezielte Aufklärung über Nutzen und Möglichkeiten rechtzeitiger Diagnostik zu Therapie, Vorsorge oder Lebensqualität ist wichtig [2]. Persönliche Gespräche und leicht verständliche Informationsmaterialien können motivieren und Ängste abbauen. Zwar lassen sich gewisse Drop-out-Raten nicht vermeiden, doch durch einen ganzheitlichen Ansatz reduzieren.

## Limitationen

Das Sampling der Interviewteilnehmenden war klein und regional begrenzt, sodass nicht alle Perspektiven abgebildet werden können. Detailtiefe der ergänzenden Dokumente variieren, was Einfluss auf ihre Qualität und Vergleichbarkeit haben kann. Da die Sekundärdaten von den Forschenden selbst erhoben wurden, können eine geringere kritische Distanz und eine unbewusste Übernahme von Interpretationsmustern vorliegen. Der enge Datenbezug stellt aber auch eine Stärke dar, da kontextspezifisches Wissen und ein vertieftes Verständnis der Erhebungssituation in die Analyse einfließen konnten [18]. Die Darstellung erhebt daher keinen Anspruch auf Vollständigkeit und fokussiert sich auf ausgewählte Hürden aus dem Projekt „iCreate“. Einstellungen zum Thema Demenz, die generelle Verfügbarkeit und Wirksamkeit verschiedener Behandlungs‑/Versorgungsformen sowie der postdiagnostische Support werden nicht weiter beleuchtet.

## Schlussfolgerung und Fazit

Das „iCreate-Projekt“ zeigt einen praxisnahen Weg auf, wie die Demenzfrüherkennung in der hausärztlichen Versorgung verbessert werden kann – durch digitale Test, Delegation von Aufgaben und Zusammenarbeit mit einer GDA. Gleichzeitig verdeutlicht es, dass vielfältige Hürden den optimalen Diagnostikprozess und eine flächendeckende Umsetzung erschweren. Eine holistische, interdisziplinäre und intersektorale Herangehensweise, die auf technische Infrastruktur, kommunikative Kompetenzen und patientenzentrierte Versorgung setzt, ist nötig. Die vorgeschlagenen Maßnahmen lassen sich hinsichtlich ihres erwarteten Effekts priorisieren. Den größten Nutzen verspricht eine verbesserte technische Infrastruktur, da hierdurch zentrale Hürden wie Brüche im Informationsfluss, unklare Abläufe und zusätzlicher Dokumentationsaufwand unmittelbar reduziert werden könnten. An zweiter Stelle steht die Standardisierung diagnostischer Verfahren, da sie vergleichbare Ergebnisse, mehr Sicherheit und Effizienz fördert und eine verlässliche Grundlage für ärztliche Entscheidungen schafft. Der Abbau von kommunikativen Hürden ist ebenfalls bedeutsam, entfaltet sein volles Potenzial jedoch voraussichtlich erst dann, wenn die strukturellen und methodischen Grundlagen zuvor gestärkt worden sind. Weitere Forschung sollte sich daher auf diese Aspekte konzentrieren, um die Diagnostik und Versorgung zu verbessern.

## Data Availability

Die erhobenen und analysierten Datensätze sind auf begründete Anfrage bei der korrespondierenden Autorin erhältlich.
